# Unravelling the molecular basis for light modulated cellulase gene expression - the role of photoreceptors in *Neurospora crassa*

**DOI:** 10.1186/1471-2164-13-127

**Published:** 2012-03-31

**Authors:** Monika Schmoll, Chaoguang Tian, Jianping Sun, Doris Tisch, N Louise Glass

**Affiliations:** 1 Plant and Microbial Biology Department, University of California, Berkeley, CA 94720, USA; 2 Research Area Gene Technology and Applied Biochemistry, Vienna University of Technology, Getreidemarkt 9, 1060 Wien, Austria; 3 Tianjin Institute of Industrial Biotechnology, Chinese Academy of Sciences, Tianjin 300308, China

## Abstract

**Background:**

Light represents an important environmental cue, which exerts considerable influence on the metabolism of fungi. Studies with the biotechnological fungal workhorse *Trichoderma reesei *(*Hypocrea jecorina*) have revealed an interconnection between transcriptional regulation of cellulolytic enzymes and the light response. *Neurospora crassa *has been used as a model organism to study light and circadian rhythm biology. We therefore investigated whether light also regulates transcriptional regulation of cellulolytic enzymes in *N. crassa*.

**Results:**

We show that the *N. crassa *photoreceptor genes *wc-1, wc-2 *and *vvd *are involved in regulation of cellulase gene expression, indicating that this phenomenon is conserved among filamentous fungi. The negative effect of VVD on production of cellulolytic enzymes is thereby accomplished by its role in photoadaptation and hence its function in White collar complex (WCC) formation. In contrast, the induction of *vvd *expression by the WCC does not seem to be crucial in this process. Additionally, we found that WC-1 and WC-2 not only act as a complex, but also have individual functions upon growth on cellulose.

**Conclusions:**

Genome wide transcriptome analysis of photoreceptor mutants and evaluation of results by analysis of mutant strains identified several candidate genes likely to play a role in light modulated cellulase gene expression. Genes with functions in amino acid metabolism, glycogen metabolism, energy supply and protein folding are enriched among genes with decreased expression levels in the *wc-1 *and *wc-2 *mutants. The ability to properly respond to amino acid starvation, i. e. up-regulation of the cross pathway control protein *cpc-1*, was found to be beneficial for cellulase gene expression. Our results further suggest a contribution of oxidative depolymerization of cellulose to plant cell wall degradation in *N. crassa*.

## Background

Light is one of the most important environmental cues for almost all living organisms. The daily rhythms of light and darkness and concomitant alterations in temperature, humidity and activity require metabolic adaptation for optimal use of cellular resources. Transmission of the light signal is accomplished by photoreceptor proteins, which are regulated and modified in response to light [1]. The filamentous fungus *Neurospora crassa *is one of the best studied organisms in this respect and has become a model system for understanding the light response and circadian rhythms [2-5]. Photoreceptors and their cognate signalling cascades have numerous regulatory targets and impact almost every aspect of physiology in *N. crassa *and other filamentous fungi [6-9]. The *N. crassa *photoreceptors White Collar 1 and 2 (WC-1 and WC-2) are transcription factors of the fungal GATA zinc finger family [5,10,11]. A complex of these proteins - the White Collar Complex (WCC) - binds to consensus GATA and LRE (light response) elements within the promoters of light regulated genes [7,12-14]. Both photoreceptors contain PAS domains, which are required for homo- and heterodimerization [10,15,16]. Strains containing loss-of-function mutations in *wc-1 *or *wc-2 *are largely blind and circadian rhythmicity is perturbed, although a minor residual response to light has been observed [8]. Genes under the control of the WCC can be circadian only, light responsive only, circadian and light responsive or neither light responsive nor circadian [17]. WC-1 and WC-2 also have individual functions besides acting as a component of the WCC [1,18,19]. VVD, the third photoreceptor of *N. crassa*, is responsible for adaptation to light [20-22] and interacts with the WCC to alter light and clock responses [23]. VVD also contains a PAS domain and acts as a negative regulator following the initiation of light response; Δ*vvd *mutants show an enhanced and prolonged response to light [8,20]. Recently, VVD was found to serve as a molecular memory of the brightness of the preceding day and to discriminate between high and low light, which is especially important for the correct reaction to moonlight [24].

Recently, it has been shown that light modulates transcription of cellulase genes in the industrially important species *Trichoderma reesei *(anamorph of *Hypocrea jecorina*) [25].

Subsequent studies aimed at the elucidation of the interconnection of light signalling and cellulase gene expression in *T. reesei *revealed that orthologs of *wc-1 *and *wc-2 *(*brl1 *and *brl2*, respectively) and components of heterotrimeric G-protein signalling are involved in light-modulated regulation of cellulase gene expression [25-29]. Light also plays a role in the sulphur requirement for growth on cellulose in *T. reesei *[30].

Transcriptome and secretome analysis highlighted the value of *N. crassa *as a model system for understanding the production of plant cell wall degrading enzymes [31]. However, the effect of light on enzyme production has not previously been evaluated in this fungus. In this study, we determined whether light regulation of transcription and production of plant cell wall degrading enzymes also occurs in *N. crassa *and how this regulation is accomplished. We show that strains containing deletion in genes encoding the photoreceptors WC-1, WC-2 and VVD influence cellulase gene expression. From transcriptional profiling data, we evaluate the phenotype of a number of mutants in genes that showed differential regulation in *wc-1, wc-2 *and *vvd *mutants as compared to wild type, revealing that these photoreceptors and the light response are involved in a complex adjustment of physiological processes and composition of the secreted enzyme mixture.

## Results

### Analysis of cellulolytic activity and growth of Δ*wc-1*, Δ*wc-2 *and Δ*vvd *mutants on cellulose

**Figure 1:**
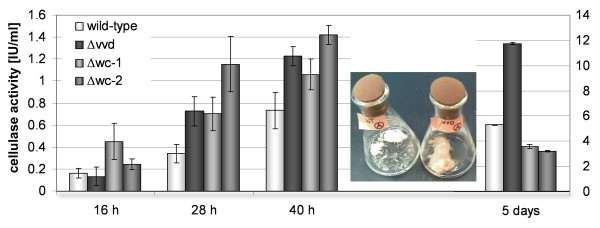
**Secretion of cellulolytic enzymes by wild type, Δ*wc-1*, Δ*wc-2 *and Δ*vvd *mutants**. Azo- CMC cellulase (endoglucanase) activity in culture filtrates of *N. crassa *wild type (WT) and mutant strains upon growth in Birds-medium with 2% (w/v) Avicel cellulose as sole carbon source at 16, 28 and 40 hrs versus 5 days. The flasks show examples for degradation of Avicel in wild type (left) and Δ*vvd *mutant (right) strains after 40 hours of growth. At this timepoint the cellulosic substrate was almost or completely degraded by the strains used in this study. Error bars show standard deviation of at least three biological replicates.

**Figure 2:**
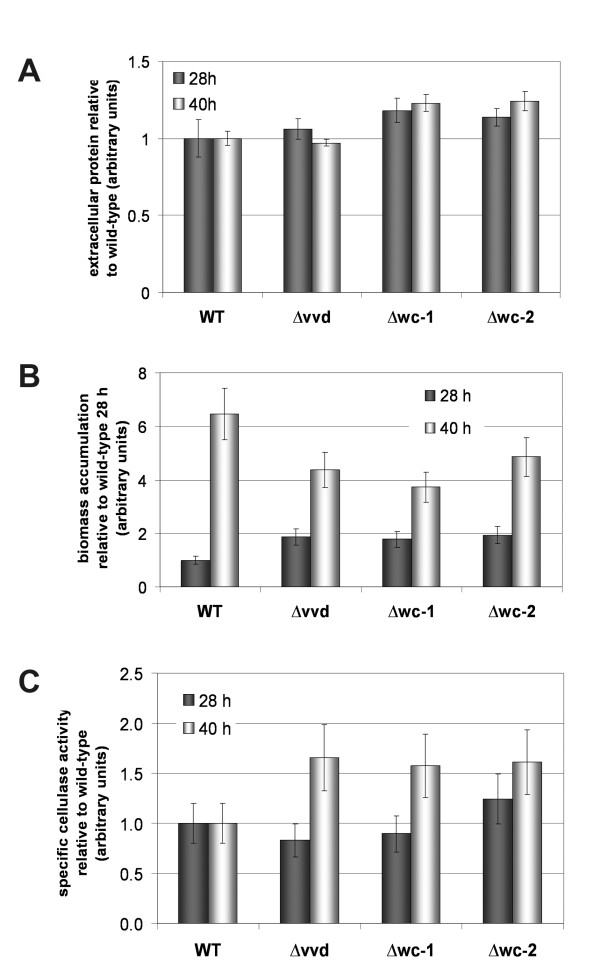
**Growth characteristics of wild type, Δ*vvd*, Δ*wc-1 *and Δ*wc-2 *mutants**. (A) extracellular protein production. The wild type strain secreted 42.7 ± 5.1 mg/L of protein after 28 hours and 51.7 ± 2.4 mg/L of protein after 40 hours of cultivation on cellulose. (B) NaOH soluble protein of mycelia reflecting biomass accumulation of WT and mutant strains after 28 or 40 hours of growth on cellulose, relative to WT at the respective time point. (C) Specific Azo- CMC cellulase activity (cellulase activity per biomass) after 28 or 40 hours of growth on cellulose relative to wild type at the respective time point. Error bars show standard deviation of at least two biological replicates.

Measurements of endoglucanase activity revealed that enzyme production is apparent after 16 hrs of growth when *N. crassa *is cultivated in liquid culture at 25°C with 2% (w/v) crystalline cellulose (Avicel) in light (Figure 1). The Δ*wc-2 *mutant showed a significantly higher level of endoglucanase activity at 28 and 40 hrs (1.15 ± 0.25 U/ml and 1.42 ± 0.08 U/ml) as compared to wild type (WT) (0.34 ± 0.08 U/ml and 0.73 ± 0.16 U/ml). However, after 5 days of cultivation on Avicel, the Δ*vvd *mutant showed significantly higher endoglucanase activity than WT (11.74 ± 0.73 U/ml versus 5.29 ± 0.36 U/ml), while the Δ*wc-1 *and Δ*wc-2 *mutants showed the lowest activity among all strains (~3 U/ml) (Figure 1). Significant differences in the amount of secreted protein among the mutant strains versus wild type were not apparent (Figure 2A, Additional file 1: Figure S1), nor were major defects in hyphal extension rates (Additional file 1: Figure S2). Whereas the Δ*wc-1*, Δ*wc-2 *and Δ*vvd *strains initially produced more biomass than the wild type (28 hrs), this difference was compensated at the later time point (40 hrs) (Figure 2B). Analysis of germination of Δ*wc-1*, Δ*wc-2 *and Δ*vvd *upon growth on cellulose did not reveal a statistically significant difference as compared to wild type germination rates or frequency (data not shown). The specific cellulase activity produced by the mutant strains was comparable to the WT during active growth (28 hrs) and increased with continued cultivation (40 hrs) (Figure 2C).

### Genome wide analysis of transcriptional regulation by photoreceptors on cellulose

**Figure 3:**
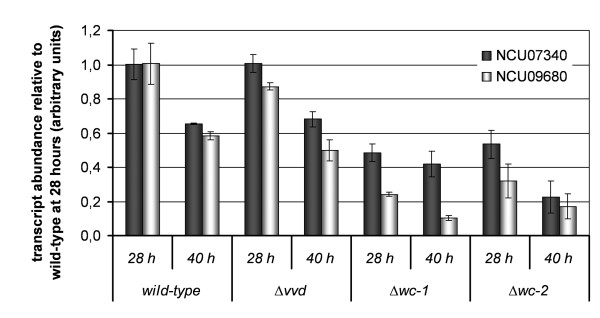
**Transcript abundance of NCU07340 (encoding CBH-1) and NCU09680 (encoding CBH-2/GH6-2) upon cultivation on cellulose**. qRT-PCR data were normalized to transcript levels in wild type after 28 hours and are given for WT after 28 (WT 28) or 40 (WT 40) hours, Δ*vvd *(VVD28 or VVD40), Δ*wc-1 *(WC1 28 or WC1 40) and Δ*wc-2 *(WC2 28 or WC2 40). *l6e *(NCU02702), a gene encoding a ribosomal protein, was used as control. Analysis was done in triplicates and error bars show standard deviation of these triplicates.

The Δ*wc-1*, Δ*wc-2 *and Δ*vvd *mutants showed alterations in cellulase activity (above), suggesting that WC-1, WC-2 and VVD might affect transcription of plant cell wall degrading enzymes. To correlate transcriptional patterns of cellulase gene expression with cellulase activity, we performed qRT-PCR of cellulolytic genes in wild type and mutant strains cultivated under the identical conditions as used for analysis of cellulase production. Taking in account both time points (28 and 40 hours), a significantly lower expression level of the *N. crassa cbh1/cel7a *homologue (NCU07340; *cbh-1*) and the *cbh2/cel6a *homologue (NCU09680; *cbh-2*) was observed in the Δ*wc-1 *and Δ*wc-2 *mutants (p-values < 0.0005; Figure 3), while expression levels of *cbh-1 *and *cbh-2 *in Δ*vvd *were comparable to the WT strain (p-values 0.934 and 0.284, respectively). To further explore how mutations in the photoreceptor genes (*wc-1, wc-2 *and *vvd*) globally affect transcriptional regulation of lignocellulolytic genes in *N. crassa*, we performed expression profiling experiments using full genome oligonucleotide microarrays constructed for *N. crassa *[32,33]. Transcriptional profiles were assessed in the mutants and wild type at two time points, when active cellulase biosynthesis was occurring (28 and 40 hrs). We chose a closed circuit design for our microarray analysis (Additional file 1 Figure S3) as it allows for robust statistical analyses [34]. Statistical support was detected for transcript levels for a total of 6154 genes (out of ~10,000) (Additional file 2: Dataset 1).

**Figure 4:**
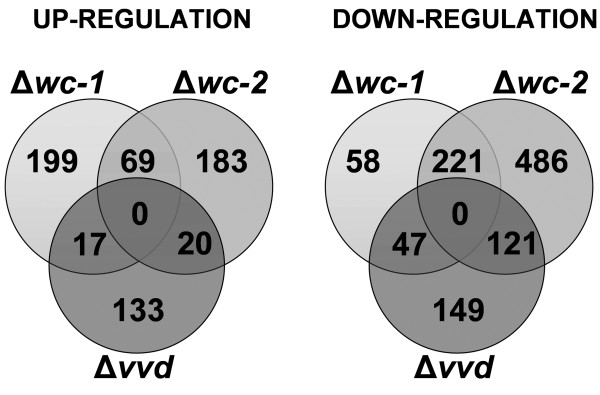
**Comparison of gene expression patterns among the three photoreceptor mutants**. (A) Venn diagram showing the overlap among genes that showed statistically significantly increase in expression levels in the respective mutant strain(s) compared to WT. (B) Venn diagram showing the overlap among genes that showed statistically significantly decrease in expression levels in the respective mutant strain(s) compared to WT.

Analysis of transcriptional patterns of WT and the photoreceptor mutants (Δ*wc-1*, Δ*wc-2 *and Δ*vvd*) revealed 1718 genes to be significantly differentially transcribed more than 1.5 fold in at least one mutant strain as compared to WT (619 genes up-regulated, 1098 down-regulated). A comparison of genes that showed increased or decreased relative expression levels in the Δ*wc-1*, Δ*wc-2 *and Δ*vvd *mutants revealed that a larger proportion of genes overlapped between the Δ*wc-1 *and Δ*wc-2 *mutants, especially in the gene set that showed decreased expression levels in the mutants (Figure 4). These data are consistent with the role of the WCC functioning in transcriptional activation. For example, WCC directly regulates photoinduction of *al-1 *(NCU00552, a phytoene desaturase; [35]) and *al-2 *(NCU00585, a phytoene synthase; [36]), which are components of the carotenoid biosynthetic pathway; *wc-1 *and *wc-2 *mutants show reduced levels of *al-1 *and *al-2 *[5,10,11], while *vvd *mutants show increased expression levels [20,37]. Consistent with these data, we observed decreased expression levels for *al-1 *and *al-2 *in Δ*wc-1 *and Δ*wc-2 *mutants and increased expression levels in Δ*vvd *(Additional file 2: Dataset 1; Additional file 3: Dataset 2). In addition, transcription data of *cbh-1 *(NCU07340) and *cbh-2 *(NCU09680) are in accordance with the data revealed by qRT-PCR, which are more precise than microarray data (Additional file 1: Figure S4).

### Gene clustering of microarray expression profiles and functional category enrichment

**Figure 5:**
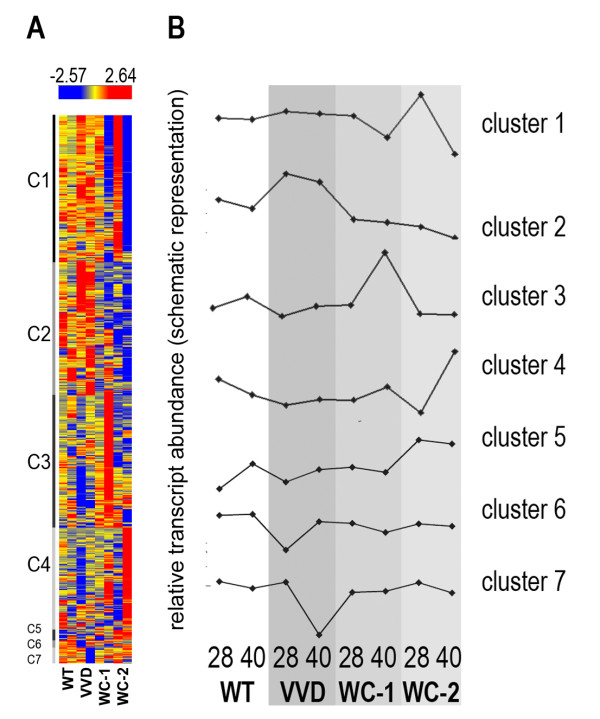
**Identification of genes with similar transcription profiles in the strains used for this study by hierarchical clustering of microarray data**. (A) A total of 1718 genes were hierarchically clustered based on their expression profiles across all strains and time points and seven clusters were identified. Normalized values are plotted as determined by HCE3.5 (B) Average expression profiles (schematic representation) of genes assigned to each cluster.

Hierarchical clustering showed that the 1718 significantly regulated genes fell into 7 clusters (Figure 5; Additional file 4: Dataset 3). Cluster 1 (461 genes) contained genes that showed reduced expression levels in both Δ*wc-1 *and Δ*wc-2 *at 40 hrs relative to both WT and Δ*vvd*. Functional category analysis showed an enrichment for metabolic genes in this cluster, especially of those involved in amino acid metabolism (P-value 6.24e-22), but also genes related to C-compound and carbohydrate metabolism (P-value 5.91e-12), including two cellulases of glycoside hydrolase (GH) family 7 (NCU04854 and *cbh-1*, NCU07340), three GH family 61 members (NCU02344, NCU02916 and NCU05969), three hemicellulases (GH family 10, NCU08189; GH family 43, NCU09652 and GH family 51, NCU02343), two β-glucosidases (NCU00130 and NCU07484) and a D-xylose reductase (NCU08384). This cluster also included early light response genes (ELRG) identified in expression profiling experiments performed under sucrose conditions [8], such as genes involved in the biosynthesis of photoprotective pigments (lipid, fatty acid and isoprenoid metabolism), vitamins, cofactors and prosthetic groups, as well as genes involved in secondary metabolism, osmotic and salt stress response. Genes responsible for energy supply, including genes encoding enzymes important in glycolysis and the tricarboxylic acid cycle, were particularly enriched in this group of genes with reduced expression levels in both Δ*wc-1 *and Δ*wc-2 *mutants, as were genes with functions in protein synthesis, transport and stress response.

The second cluster (cluster 2; 417 genes) contained genes that showed increased expression levels in Δ*vvd*, but with decreased expression levels in the white-collar mutants (Figure 5). This cluster showed significant enrichment of genes involved in C-compound and carbohydrate metabolism (P value = 3.54e-14). Genes in this category included the GH6 family cellulases (NCU09680 and NCU07190), a GH7 family cellulase (NCU05057), 6 GH61 family cellulases (NCU00836, NCU02240, NCU03328, NCU07760, NCU07898, NCU08760) as well as four putative acetyl xylan esterases (NCU00710, NCU04870, NCU05159 and NCU09664) and one cellobiose dehydrogenase (NCU00206). Additionally, a slight enrichment of genes involved in oxygen and radical detoxification (P value = 5.70e-4), such as the catalase NCU00355, was observed.

Genes assigned to cluster 3 (415 genes) showed a large increased expression level specifically in the Δ*wc-1 *mutant at 40 hrs (Figure 5). This cluster was predominantly enriched in genes involved in phosphate metabolism (P value = 9.68e-04), energy metabolism (P value = 7.49e-05), ATP binding (P value = 7.76e-06) and stress response (P value = 1.64e-04). Genes assigned to cluster 4 (320 genes) showed an increase in expression level at 40 hrs, particularly in the Δ*wc-2 *mutant (Figure 5). The functional category of polysaccharide metabolism was significantly enriched in this cluster, albeit to a lower extent than clusters 1 and 2 (P value = 1.52e-4). Interestingly, this group comprised three GH 2 family genes (NCU06781, NCU07253 and NCU08909), which encode a β-galactosidase, a β-mannosidase and a β-glucuronidase, respectively. Genes included in clusters 5 and 6 (32 and 21 genes respectively) did not show an enrichment for any functional category, while genes in cluster 7 (51 genes) showed a strong decrease in transcript abundance in the Δ*vvd *mutant at 40 hrs and were enriched in the functions of respiration (P value = 3.55e-5) and transport of substrates (P value 6.62e-4).

### Specific regulation in individual photoreceptor mutants

**Figure 6:**
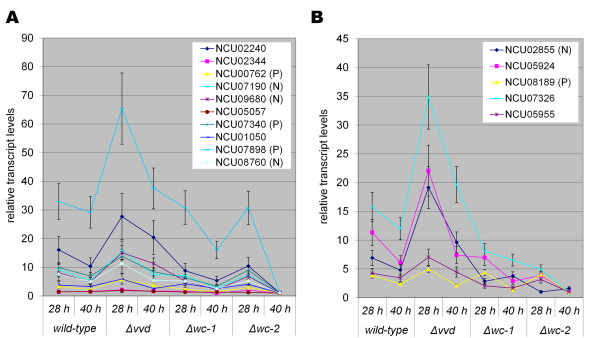
**Transcript abundance of cellulase and hemicellulase genes in wild type and mutant strains upon growth on cellulose**. (A) Genes encoding cellulases for which their contribution to cellulase activity has been evaluated [31], and this study. (B) Genes encoding hemicellulases for which their contribution to cellulase activity has been evaluated [31]. The suffix (P) indicates positive effect on efficiency of the secreted cellulase mixture, (N) indicates a negative effect on the secreted cellulase mixture. (C) Hierarchical clustering of gene regulation patterns in wild type and mutant strains as analyzed using HCE3.5 with the average linkage (UPGMA) method and the Pearson correlation coefficient as distance measure.

To obtain detailed information on the specific function of the individual photoreceptors under cellulolytic conditions, we analyzed and compared expression profiles for each mutant individually to WT (Table 1 Additional file 3: Dataset 2). In the Δ*wc-1 *mutant, six cellulase genes (NCU02344, NCU04854, NCU05969, NCU7340, NCU7760, NCU9680), four hemicellulase genes (NCU02343, NCU05924, NCU08189, NCU09652) as well as three putative β-glucosidase genes of GH families 1 and 3 (NCU00130, NCU04952, and NCU07487) were significantly down-regulated (P values < 0.005; Figure 6A, B). Additionally, significantly reduced expression levels for genes involved in amino acid metabolism (P value = 8.13e-13), translation (P value = 8.17e-04), protein folding and stabilization (P value = 1.19e-06) as well as energy supply (P value = 5.36e-21) (Additional file 3: Dataset 2) were observed. Among genes with increased expression level in the Δ*wc-1 *mutant, only an enrichment for C-3 compound metabolism was observed (P value = 3.84e-04).

**Table 1:** Transcript levels of cellulase, hemicellulase and beta-glucosidase genes in photoreceptor mutants

cellulases	GH family	Δ*wc-1*	Δ*wc-2*	Δ*vvd*
NCU00762	5		down	up
NCU03996	6			
NCU07190	6		down	up
NCU09680	6	down	down	
NCU04854	7	down	down	
NCU05057	7		down	
NCU05104	7			
NCU07340	7	down	down	
NCU05121	45			
NCU00836	61		down	
NCU01050	61		down	
NCU01867	61			
NCU02240	61		down	up
NCU02344	61	down	down	up
NCU02916	61		down	
NCU03000	61			
NCU03328	61		down	
NCU05969	61	down	down	
NCU07520	61			
NCU07760	61	down		
NCU07898	61		down	
NCU07974	61			
NCU08760	61		down	

**hemicellulases**	**GH family**	**Δ*wc-1***	**Δ*wc-2***	**Δ*vvd***

NCU05924	10	down	down	up
NCU08189	10	down	down	
NCU04997	10			
NCU07130	10			
NCU02855	11	down	down	up
NCU07225	11			
NCU08087	26			
NCU07326	43		down	up
NCU01900	43			
NCU05965	43			
NCU09170	43			
NCU09652	43	down	down	
NCU00852	43			
NCU06861	43			
NCU02343	51	down	down	
NCU00972	53			
NCU09775	54			
NCU07351	67		down	
NCU05955	74		down	

**beta-glucosidases**	**GH family**	**Δ*wc-1***	**Δ*wc-2***	**Δ*vvd***

NCU00130	1	down	down	
NCU03641	3			
NCU04952	3	down	down	
NCU07487	3	down	down	
NCU08054	3			

The decreased expression levels of genes involved in plant cell wall degradation observed in the Δ*wc-1 *mutant were even more pronounced in the Δ*wc-2 *mutant (Table 1), with additional genes encoding cellulolytic enzymes showing decreased expression levels, including the predicted cellulases NCU00836, NCU01050, NCU02240, NCU02916, NCU03328, NCU05057 and NCU07190 as well as the hemicellulases NCU05955 and NCU07351 (Figure 6A, B; Additional file 1: Figure S5), for a total of 15 cellulase genes (out of 23 predicted;) and 8 hemicellulase genes (out of 19 predicted) [31]. From the cellulases known to be secreted into the medium [31] and hence most likely to contribute to the efficiency of the cellulolytic enzyme mixture of the mutants, two were significantly down-regulated in both Δ*wc-1 *and Δ*wc-2 *(NCU07340 and NCU09680), which correspond to *cbh-1 *and *cbh-2*, respectively (Figure 3). Deletion of either *cbh-1 *or *cbh-2 *affects cellulolytic activity [31]. Of the four secreted hemicellulases (NCU05924, NCU05955, NCU07326 and NCU08189 [31]), two (NCU05924, NCU08189 encoding an endo-1,4-beta xylanase) were found to be significantly down-regulated in both white collar mutants, while NCU07326 (encoding a glycoside hydrolase family 43) and NCU05955 were down-regulated only in Δ*wc-2*. In Δ*vvd*, NCU05955 and NCU07326 were up-regulated. However, up to ~72 hr of growth, the spectrum and amount of protein secreted into the culture filtrate did not show major defects in the Δ*wc-2 *mutant, even though gene expression profiles for many secreted hydrolases were reduced (Figure 2A, Additional file 1: Figure S1).

In addition to an enrichment for genes within the functional category C-compound and carbohydrate metabolism that showed decreased expression levels in Δ*wc-2 *(P-value 3.67e-25), expression levels for genes encoding proteins involved in protein synthesis (P value = 2.45e-35), energy supply (P value = 1.31e-19) and amino acid metabolism (P value = 6.44e-14) were decreased. Genes involved in secretion were not affected in expression level in the white collar mutants, in agreement with earlier data on cellulase expression in *T. reesei*, for which also no enhancement of secretion was found under cellulase inducing conditions or in high performance mutants [38].

Among the genes that showed increased expression levels in the Δ*wc-2 *mutant, genes with functionalities of lysosomal and vacuolar protein degradation (P-value 3.03e-4) as well as polysaccharide degradation (P-value 4.95e-5) were significantly enriched. In addition, a few genes related to polysaccharide metabolism showed increased expression levels, for example GH family 2 genes NCU06871, NCU07253 and NCU08909, a glycoside transferase NCU08226 and a pectate lyase NCU09791; no predicted cellulase or hemicellulase genes were identified.

In the Δ*vvd *mutant, the gene set that showed 1.5 fold significant increase in expression level included those responsible for C-compound and carbohydrate degradation, such as four cellulases (NCU00762, NCU02240, NCU02344 and the most highly produced endoglucanase in *N. crassa *- NCU07190) and three hemicellulases (NCU02855, NCU05924 and NCU07326) (Table 1). Of these genes, NCU00762 [39] encodes a secreted endoglucanase (*gh5-1*) [31,40]; deletion of NCU00762 significantly reduced endoglucanase activity in *N. crassa *[31].

Among the down-regulated genes in the Δ*vvd *mutant, enrichment for functions in energy supply (P value 9.65e-05) and phosphate metabolism (P value 5.81e-04) was observed. With respect to genes within C-compound and carbohydrate metabolism, we also found a decrease in transcription of the genes involved in transport (for example of carboxylic acid, glycerol or hexoses; P value = 5.78e-03) and metabolism of energy reserves (for example glycogen synthase; P value = 1.31e-03). Thus, the expression patterns observed in the Δ*wc-1 *and Δ*wc-2 *mutants are more similar to each other, especially in the reduced expression levels of genes within the C-compound and carbohydrate metabolism functional category, while an opposite pattern was observed in the Δ*vvd *mutant. This conclusion is also reflected upon hierarchical clustering of expression patterns (Figure 6C). Additionally this analysis showed the increasing importance of WC-1 and WC-2 after longer cultivation times on cellulose. However, our transcriptional data suggests that the function of WC-1 and WC-2 is not exerted solely as the WCC towards the transcriptional response to growth on cellulose, but that WC-2, especially, has a more important effect on expression patterns and physiology than WC-1.

### Identification of genes regulated by WC-1 or WC-2 versus the WCC

Because of the expression differences in the Δ*wc-1 *or Δ*wc-2 *mutants, we evaluated whether genes sets were regulated (directly or indirectly) by the WCC-complex (similar regulation in both mutants) or regulated (directly or indirectly) by WC-1 or WC-2 individually, but not by the WCC (Additional file 5 Dataset 4). Direct targets of WCC have previously been identified by chromatin-immunoprecipitation under sucrose grown conditions [7]. We identified a 27-gene overlap (in the total dataset of 290 genes showing differences in expression level in both Δ*wc-1 *and Δ*wc-2 *mutants versus wild type) to be direct targets of WCC (Additional file 5: Dataset 4). Besides several metabolic genes (a chitinase NCU07035, a GH family 2 protein NCU08909 and a pectate lyase NCU09791), these overlapping targets include *vvd *[20,41], the carbon catabolite repressor gene *cre-1 *(NCU08807), two genes involved in conidiation (*csp-1 *and *con-8*) and a hydrophobin gene (*ccg-2*). Cellulases or hemicellulases were not identified among the direct targets of WCC upon growth on sucrose [7].

Our data suggest that WC-1 and WC-2 may also have independent functions. We consequently analyzed which functionalities were affected individually in expression profiles from the *wc-1 *or *wc-2 *photoreceptor mutants but not by the WCC. Within the WCC regulated gene set, 221 genes showed decreased expression levels, while 69 genes showed increased expression levels (Figure 4; Additional file 5: Dataset 4). In the Δ*wc-1 *mutant, 105 genes showed decreased expression levels and 216 genes showed increased expression levels. In the Δ*wc-2 *mutants, 607 genes showed reduced expression level, while 203 genes showed increased expression levels (Figure 4; Additional file 5: Dataset 4). Metabolic functions including C-compound and carbohydrate metabolism (Δ*wc-1 *P-value = 2.17e-08; Δ*wc-2 *P value = 5.00e-09) were strongly down-regulated in these strains individually as were genes contributing to energy supply (Δ*wc-1 *P-value = 1.89e-07; Δ*wc-2 *P value = 7.54e-08). These functionalities were also enriched in putative WCC-targets, but the individual regulation of components of these pathways indicates additional and distinct roles of WC-1 and WC-2 in these processes.

**Figure 7:**
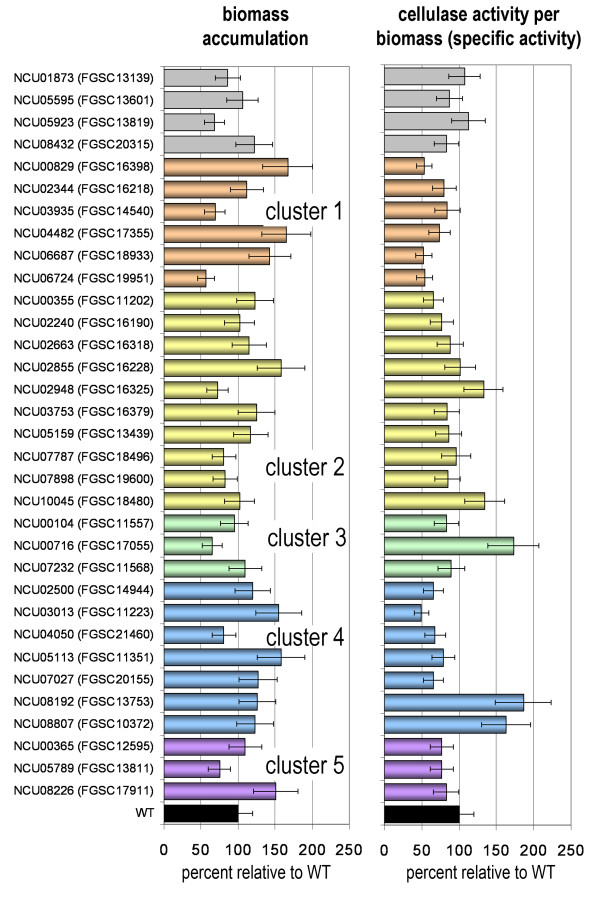
**Analysis of selected mutant strains lacking genes with expression profiles or functions of interest for cellulase gene expression**. Strains as described in Table 2 are presented according to the clusters assigned to the respective gene. Designation of strains is valid for both columns. Cultivation of strains was performed on Avicel cellulose (2% w/v) for 40 hrs. Specific cellulase activity (cellulase per biomass) and biomass content were determined and normalized to WT. Every measurement was done with at least two biological and two technical replicates. Eight biological replicates were considered for wild type. Error bars show standard deviation of the measurements considering all biological replicates.

In the Δ*wc-2 *mutant, the unfolded protein response pathway was significantly down-regulated (P value = 1.66e-04). The most striking difference, however, was the considerable enrichment of the functionalities of ribosome biogenesis, ribosomal proteins and translation (P-values < 1e-33) among genes that showed decreased expression levels in the Δ*wc-2 *mutant only. In the gene set that showed increased expression levels in the Δ*wc-2 *mutant, nine genes involved in polysaccharide metabolism (which are not increased in Δ*wc-1*) were identified, including a gene encoding a predicted glycosyl transferase family 2 enzyme (NCU08226) and a putative GH16 enzyme (NCU05789) (Table 2). Both genes are members of cluster 5 (Figure 5). We tested specific cellulase activity in strains carrying deletions of NCU08226 or NCU05789 (Figure 7). However, both mutants were not significantly different than WT, although lack of NCU08226 caused significantly increased biomass production.

**Table 2:** Genes selected for analysis with respect to cellulase expression and deletion strains

Strain	locus ID	description/function	topic/selection criteria	potential contribution to cellulolytic efficiency in
FGSC12595	NCU00365	hypothetical protein, repressed by CPC-1	amino acid metabolism	
FGSC14540	NCU03935	homoserine dehydrogenase	amino acid metabolism	
FGSC21460	**NCU04050**	*cpc-1 *encoding cross pathway control protein	amino acid metabolism	Δ*wc-1 *and *Δwc-2*
FGSC17355	**NCU04482**	hypothetical protein, upregulated by aa starvation	amino acid metabolism	Δ*wc-1 *and *Δwc-2*
FGSC19959	**NCU06724**	glutamine synthetase	amino acid metabolism	
FGSC14944	**NCU02500**	*ccg-4 *alpha type peptide pheromone	consistent regulation	
FGSC18933	**NCU06687**	glycogen synthase	consistent regulation/glycogen metabolism	
FGSC20155	**NCU07027**	glycogen phosphorylase	consistent regulation/glycogen metabolism	
FGSC17911	**NCU08226**	glycosyl transferase family 2	consistent regulation/glycogen metabolism	Δ*wc-2*
FGSC11557	NCU00104	heat shock protein, HSP98-like	differential regulation by WC-1 and WC-2	
FGSC17055	**NCU00716**	non-anchored cell wall protein, *ncw-5*	differential regulation by WC-1 and WC-2	Δ*vvd *and Δ*wc-2*
FGSC11568	NCU07232	heat shock protein 30, light responsive	differential regulation by WC-1 and WC-2	
FGSC11202	**NCU00355**	catalase-3	fenton chemistry/oxidative depolymerization	Δ*vvd*
FGSC16398	**NCU00829**	ferric reductase	fenton chemistry/oxidative depolymerization	Δ*vvd*
FGSC13139	NCU01873	cellobiose dehydrogenase	fenton chemistry/oxidative depolymerization	
FGSC16325	NCU02948	quinone oxidoreductase, type IV; light responsive; non-anchored cell wall protein, *ncw-4*	fenton chemistry/oxidative depolymerization	
FGSC11223	**NCU03013**	copper/zinc super oxide dismutase (SOD); anchored cell wall protein, *acw-10*	fenton chemistry/oxidative depolymerization	
FGSC11351	**NCU05113**	multicopper oxidase/laccase precursor	fenton chemistry/oxidative depolymerization	Δ*wc-2*
FGSC13601	NCU05595	cellobiose dehydrogenase	fenton chemistry/oxidative depolymerization	
FGSC13819	**NCU05923**	cellobiose dehydrogenase	fenton chemistry/oxidative depolymerization	
FGSC20315	NCU08432	cellobiose dehydrogenase	fenton chemistry/oxidative depolymerization	
FGSC10372	NCU08807	carbon catabolite repressor *cre-1*	target of the white collar complex	
FGSC16190	NCU02240	endoglucanase II, glycosyl hydrolase family 61	up-regulation in Δ*vvd*	
FGSC16218	NCU02344	cellulose binding protein CEL1, glycoside hydrolase family 61	up-regulation in Δ*vvd*	
FGSC16318	NCU02663	L-lysine 2,3 aminomutase; radical SAM superfamily	up-regulation in Δ*vvd*	
FGSC16228	**NCU02855**	endo 1,2 beta xylanase A; glycosyl hydrolase family 11	up-regulation in Δ*vvd*	Δ*vvd*
FGSC16379	NCU03753	*grg-1 *encoding glucose repressible protein; clock controlled protein CCG-1	up-regulation in Δ*vvd*	
FGSC13439	NCU05159	acetylxylan esterase, contains fungal cellulose binding domain	up-regulation in Δ*vvd*	
FGSC18946	NCU07787	clock controlled gene *ccg-14*, probable Snodprot1, ceratoplatanin like	up-regulation in Δ*vvd*	
FGSC19600	NCU07898	endoglucanase IV, glycosyl hydrolase family 61	up-regulation in Δ*vvd*	
FGSC18480	NCU10045	pectin esterase	up-regulation in Δ*vvd*	
FGSC13753	**NCU08192**	fungal hydrophobin, magnaporin related	up-regulation in Δ*wc-1 *and *Δwc-2*	
FGSC13811	NCU05789	endo 1,3 (4) beta glucanase, glycosyl hydrolase family 16	up-regulation in Δ*wc-2*	

Strains are ordered according to the criteria for which they were selected for analysis. Selection criteria correspond to the topics important for evaluation of cellulase production in the respective mutant. The result of this evaluation was used to determine whether this gene may contribute to the efficiency of the cellulase mixture of **Δ***wc-1*, **Δ***wc-2 *or **Δ***vvd*. Mutants were checked for known growth defects http://www.broadinstitute.org/annotation/genome/neurospora/MultiHome.html. Gene loci given in bold cause significantly altered specific cellulase activity or biomass accumulation on cellulose. Where available, data showed no indication of growth defects in the mutant strains used in this study

### Impact of WC-1, WC-2 and VVD on known cellulase regulating genes

The carbon catabolite repression factor *cre-1 *[42,43] is subject to light-regulation by the WCC [7,8], as is the cross pathway control regulator *cpc-1 *[32], which plays an important role in regulation of amino acid metabolism. In *T. reesei*, cellulase regulation is affected by homologues of the HAP-complex [44], the transcriptional activator *xyr1 *[45] and repressor *ace1 *[46]. While neither the HAP-complex homologues nor the *xyr1 *homologue showed altered transcriptional patterns in the *N. crassa *photoreceptor mutants as compared to wild type, an *ace1 *homolog (NCU09333) showed increased expression levels in the Δ*wc-2 *mutant after 40 hrs. Similarly, transcriptional levels of *cpc-1 *and *cre-1 *increased in both white-collar mutants after 40 hrs (Additional file 2: Dataset 1).

Genomic and functional analyses of the high cellulase producing *T. reesei *mutant strain RutC30 showed that the lack of functional CRE1 is an important factor in increased cellulase activity [47-49]. In *N. crassa*, a deletion of *cre-1 *(NCU08807 [43]) resulted in an increase of 63 ± 14% in biomass-specific cellulase activity compared to WT (Figure 7). Of the 72 genes altered or missing in RutC30 [47-49], 12 were detected in our analysis, of which 8 showed at least a 1.5-fold difference in regulation in at least in one mutant strain. Among them were two genes belonging to cluster 3 (increased expression level in Δ*wc-1*) encoding a MFS multidrug transporter, NCU00306 and a transcription factor, NCU00289 and one gene in cluster 4 (increased expression level in Δ*wc-2*) encoding a predicted nitrilase, NCU01838. The remaining 5 genes (NCU02771, uroporphyrinogen decarboxylase; NCU01332, a vacuolar membrane ATPase; NCU06294, hypothetical protein; NCU06961, an exopolygalacturonase belonging to glycosyl hydrolase family 28 and NCU07129, a predicted amino acid permease) showed increased expression levels in the Δ*vvd *and decreased expression levels in the white-collar mutants (cluster 2).

Interestingly, we found that transcription of NCU05137, encoding a highly conserved non-anchored cell wall protein (NCW-1), the deletion of which causes significantly enhanced secretion of cellulolytic enzymes [31,50], was co-regulated with the known carbon catabolite repression protein encoding *cre-1 *(NCU08807) and the cross-pathway control protein encoding *cpc-1 *(NCU04050), which impacts the expression of a large number of target genes [32]. These observations suggest that NCU05137 encodes an auxiliary secreted/cell wall protein important for modulation of extracellular cellulose degrading capacity.

### Genes likely to contribute to altered cellulose degradation in photoreceptor mutants

Previously, 14 mutants in genes encoding proteins secreted under cellulolytic conditions were evaluated for cellulase activity [31]; supernatants from most mutants showed near WT cellulolytic activity, with the exception of *Δcbh-1 *(NCU07340; cellobiohydrolase), Δ*gh6-2 *(NCU09680; cellobiohydrolase), Δ*gh5-1 *(NCU00762; endoglucanase), Δ*gh3-4 *(NCU04952; β-glucosidase) and *ncw-1 *(NCU05137; cell wall associated protein of unknown function [50]). We selected mutants in three additional genes encoding GH family 61 enzymes (NCU02240, NCU07898 and NCU02344) and one GH family 11 hemicellulase (NCU02855) belonging to cluster 2 (up in Δ*vvd*) for further analysis; no significant alteration in specific cellulase gene expression was observed for mutants in any of the three GH 61 family cellulases (Figure 7). However, deletion of NCU02855 (endo-1,2-beta xylanase A) resulted in considerably increased growth on cellulose (Figure 7) and cellulase secretion, albeit specific cellulase activity was not increased. Among other genes assigned to cluster 2, we also identified acetyl xylan esterases, which have been reported to enhance the performance of *T. reesei *CBH1/Cel7a [51]. However, a strain carrying a deletion of NCU05159, which encodes an acetyl xylan esterase, did not significantly affect specific cellulase activity, while a mutant in NCU10045 (encoding pectin esterase), showed only a slight increase in specific cellulase activity (Figure 7).

Genes encoding hydrophobins have been reported to be regulated by photoreceptors in *T. atroviride *[52] and by light in *T. reesei *[28,53]. Although hydrophobins are not known to have a major influence in cellulase efficiency [54], deletion of the strongly regulated NCU08192 (related to the *M. grisea *hydrophobin MHP1 [55], cluster 4), that showed increased expression levels in both white collar mutants, unexpectedly had significantly increased specific cellulase activity (Figure 7). A strain containing a mutation in another hydrophobin-like protein belonging to cluster 2 (*ccg-14*/NCU07787), encoding a ceratoplatanin-like small protein [56,57] displayed cellulolytic activity indistinguishable from WT (Figure 7). An additional gene in cluster 2, NCU02663, encoding an L-lysine 2,3 aminomutase, showed an increase in relative expression of ~50-fold in the Δ*vvd *mutant compared to the white-collar mutants. However, lack of NCU02663 (FGSC16318), did not result in a significant alteration in cellulase activity (Figure 7).

From cluster 3 (genes with increased expression level in Δ*wc-1*), two putative heatshock proteins (NCU00104 and NCU07232) and a gene encoding an uncharacterized cell wall bound protein [50] (NCU00716), were selected for further analysis. Strains containing deletions of NCU00104 and NCU07232 displayed WT cellulase activity. However, deletion of the hypothetical protein encoding NCU00716 caused a significant increase in specific cellulase activity (Figure 7). Since this gene was clearly down-regulated in Δ*vvd *and Δ*wc-2*, a contribution to enhanced cellulase efficiency in these strains is possible.

Only three genes were found to have significantly reduced expression levels in Δ*wc-1*, Δ*wc-2 *and Δ*vvd*, albeit in part less than 1.5 fold. One strongly down regulated gene encoded the alpha type peptide pheromone precursor PPG-1 (*ccg-4*; NCU02500, cluster 4); a Δ*ccg-4 *mutant showed a slight decrease in cellulase activity (Figure 7). Two additional genes were predicted to be involved in glycogen metabolism: NCU02797, encoding a predicted UTP glucose-1-phosphate uridylyltransferase and NCU06687, encoding glycogen synthase. Since a link between glycogen metabolism and cellulase production has been proposed in *T. reesei *[27,58], we evaluated the cellulolytic phenotype of a ΔNCU06687 mutant (cluster 1); a significant reduction in both biomass and cellulase activity was observed (Figure 7). To further test for the relevance of glycogen metabolism in cellulase production, we also analyzed the cellulolytic activity of a strain lacking the gene for the catabolic enzyme glycogen phosphorylase *gph-1 *(NCU07027) (cluster 4). Similar to the glycogen synthase mutant (ΔNCU06687), specific cellulase activity was significantly reduced in the ΔNCU07027 mutant (Figure 7).

### Co-regulation of genes with *cbh-1 *(NCU07340)

The glycoside hydrolase family 7 cellulase CBH-1 (NCU07340) is a homologue of *T. reesei *Cel7a/CBH1, which is the major cellulase in this fungus [59]. Deletion of *cbh-1 *causes the most severe effect among cellulase genes in terms of both reduced growth on cellulose and decreased cellulase production in *N. crassa *[31]. We therefore assessed co-regulation of genes with *cbh-1 *in WT versus the photoreceptor mutants to interconnect regulation of cellulose degradation and the light response. Hierarchical clustering of expression data revealed 70 genes that showed co-regulation with *cbh-1 *(Additional file 4: Dataset 3). Among these genes we found one additional cellulase besides *cbh-1 *(NCU02344), one hemicellulase (NCU09652), one beta-glucosidase (NCU07487), a mannosyltransferase (NCU07338) and the aldose epimerase GAL10 (NCU09705). Interestingly, besides several amino acid biosynthetic genes in this cluster (NCU01300, NCU01666, NCU06724, NCU08409 and NCU09320), we also found a sulphate adenylyltransferase (NCU01985). This gene is required for sulphate metabolism, which was shown to be important for cellulase gene expression in *T. reesei *[30]. This finding supports the relevance of sulphur metabolism for cellulase gene expression to be conserved also in *N. crassa*.

Functional category analyses of these 70 genes showed significant enrichment in C-compound and carbohydrate metabolism (P value = 9.61e-04), as expected, but also amino acid metabolism (P value = 9.76e-04), which may play a role in cellulose degradation due to its specific regulation in the photoreceptor mutants. Additionally, we found genes responsible for protein synthesis (P value = 4.91e-04) and translation to be enriched among these 70 genes, which suggests posttranslational regulation and/or modification of cellulose degrading enzymes. The enrichment of mitochondrial functions (P value = 2.12e-04) is in accordance with earlier findings on the importance of the physiological state of the mitochondria for cellulase gene expression [60].

Within this 70-gene cluster, we evaluated cellulose activity in two additional deletion strains, one lacking NCU00829, a ferric reductase and a ΔNCU06724 (glutamine synthetase) mutant. Both strains showed a significant decrease in specific cellulase activity (Figure 7). Interestingly, NCU00829, a putative ferric reductase, also showed an increase in relative expression level in the Δ*vvd *mutant (Additional file 2: Dataset 1), suggesting a contribution of oxidative depolymerization of cellulose in *N. crassa*, which may be important for the efficient cellulose degradation.

### Involvement of oxidative depolymerization and Fenton chemistry in degradation of cellulose by *N. Crassa*

The transcriptional pattern of cellulose degrading enzymes in the white collar mutants as well as the positive effect of the ferric reductase encoded by NCU00829 on cellulase activity led us to hypothesize that fenton chemistry, in which an oxidative process characterized by the formation of highly reactive hydroxyl radicals by reaction of Fe(II) and H_2_O_2_, might contribute to cellulose depolymerization, as has been suggested for the basidiomycete *Postia placenta *[61]. We therefore analyzed the transcriptional patterns of genes putatively involved in this process such as ferric reductases, multicopper oxidases and GMC oxidoreductases (Additional file 1: Table S1) and selected genes encoding different functionalities (ferric reductase, catalase, quinone oxidoreductase, multicopper oxidase, copper superoxide dismutase) putatively involved in oxidative depolymerization of cellulose or its regulation [61] for further analysis. The selected genes were significantly regulated more than 1.5 fold in at least one of the photoreceptor mutant strains and clustered with genes up-regulated in Δ*vvd *(cluster 2) and included NCU00829 (ferric reductase; see above), NCU00355 (catalase-3), NCU02948 (*ncw-4*, encoding the type IV quinone oxidoreductase), NCU03013 (predicted copper superoxide dismutase anchored cell wall protein, *acw-10*) and NCU05113 (multicopper oxidase). Proteins encoded by NCU00355, NCU02948 and NCU03013 were reported to be associated with the cell wall in *N. crassa *[50]. Specific cellulase activity decreased significantly in strains containing deletions of NCU00355 and NCU03013, while a strain lacking NCU02948 (cluster 2) did not differ significantly from wild type (Figure 7). A strain containing a deletion of NCU05113 did not show a statistically significantly altered specific cellulase activity, but showed a considerably increased biomass formation upon growth on cellulose (Figure 7). Since deletion of three of the five genes led to a decrease in specific cellulase activity, fenton chemistry may take part in the attack on cellulose in *N. crassa*.

Recent reports showed contributions of a cellobiose dehydrogenase and a GH61 protein to oxidative cleavage of cellulose microfibrils [62-65]. We identified a cellobiose dehydrogenase (NCU00206) belonging to cluster 2 [65,66]. Unfortunately, only a heterokaryotic deletion strain (FGSC16368) was available. To evaluate whether other probable/predicted cellobiose dehydrogenases (CBDH) affected cellulase activity, we analyzed a homokaryotic strain lacking a probable CBDH (NCU05923) and three homokaryotic mutants with carrying deletion of a gene related to CBDH proteins (NCU01873, NCU05595 and NCU08432). However, none of these mutants affected cellulase activity, although the strain carrying a deletion of NCU05923 caused significantly decreased biomass production on cellulose (Figure 7) suggesting a function beneficial for growth on cellulose.

### Assessment of the role of amino acid metabolism

Transcriptional profiling analyses suggested that genes involved in amino acid metabolism may be regulated by WC-1 and WC-2 during growth on cellulose. This regulation has already been reported for growth on glucose [8] and may thus represent a general phenomenon correlated to the light response. A study with the *T. reesei *mutant RutC30 showed that decreased ability to utilize amino acids correlates with increased cellulase gene expression [48]. Additionally, an interrelationship between methionine- and sulphur signalling as well as a clear difference of the respective effect in light and darkness has been shown for *T. reesei *[30]. Since the response to amino acid starvation is mainly regulated by CPC-1 in *N. crassa *[32,67], which directly or indirectly targets more than 400 genes [32], we hypothesized that carbohydrate metabolism on cellulose might also be a target of CPC-1. Consequently, we compared the CPC-1 targets [32] with the genes regulated by both WC-1 and WC-2 (290 total). Indeed, 48 genes were found to be targets of both CPC-1 and WCC. Functional analysis of these 48 genes revealed significant enrichment in functionalities in both amino acid metabolism (P value = 1.99e-06) and C-compound and carbohydrate metabolism (P value = 7.10e-04) (Additional file 6: Dataset 5). These data support the hypothesis that the light signalling pathway is interconnected with amino acid metabolism with one of the output targets being cellulase gene expression.

To test this hypothesis, we evaluated the cellulolytic phenotype of mutants in genes involved in regulation of amino acid metabolism. The transcription of *cpc-1 *(NCU04050), [32,67] was enhanced in the Δ*wc-1 *and Δ*wc-2 *mutants. A strain lacking *cpc-1 *showed significantly decreased cellulase activity and lower biomass formation on cellulose (Figure 7). Similarly, strains carrying deletions of genes identified within the CPC-1 regulon [32], NCU04482 and NCU00365 (both hypothetical proteins) caused a slight reduction in specific cellulase activity (Figure 7). As noted above, a ΔNCU06724 mutant (encoding a glutamine synthetase and co-regulated with NCU07340; Additional file 4: Dataset 3) showed significantly decreased specific cellulase activity (Figure 7), while a strain carrying a deletion of NCU03935, encoding a homoserine dehydrogenase (cluster 1) was not significantly different from WT. Other genes shown to be responsive to amino acid starvation ([32]; Fungal Gene Expression Database: http://bioinfo.townsend.yale.edu/index.jsp) and regulated in the photoreceptor mutants showed a significant decrease in specific cellulase activity (NCU07027, glycogen phosphorylase and NCU00355, catalase, see above and Figure 7). An additional member of this cluster, NCU03753 (glucose repressible protein, *ccg-1*), showed a slight decrease in specific cellulase activity. These results suggest a link between response to amino acid starvation and modulation of cellulase gene expression in *N. crassa *as well as in *T. reesei*.

## Discussion

The preferred natural habitats of both *Trichoderma *and *Neurospora *are humid tropical and subtropical forests [68,69] and hence utilization of cellulosic substrates is crucial in their life style. Using resources available for *N. crassa *together with extensive information about cellulose degradation in *T. reesei *[68,70,71], we aimed in this study to obtain a more detailed understanding of the physiology of cellulase gene expression across fungi. We show that photoreceptors not only modulate cellulase gene expression in *T. reesei *[25,26,72] but also in *N. crassa*. Moreover, the resulting effect seems to be largely similar in that transcript levels of the major cellulases tend to be decreased in strains lacking the photoreceptors, while secreted levels of cellulolytic enzymes are increased. Therefore, we conclude that light-modulated expression of cellulolytic enzymes is a conserved phenomenon, most probably providing an evolutionary advantage to filamentous fungi in their natural habitat.

**Figure 8:**
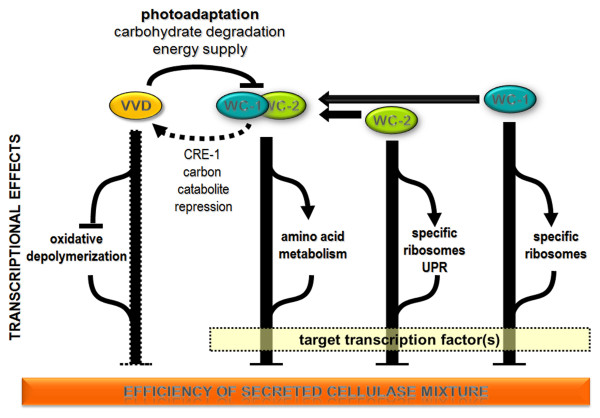
**Model for regulation of plant cell wall degrading capacity in *N. crassa *by WC-1/WC-2/WCC and VVD**. VVD, WC-1 and WC-2 negatively affect efficiency of cellulose degradation during early stages of growth. Regulation of cellulolytic enzymes and energy supply is subject to photoadaptation by VVD, while carbon catabolite repression as mediated by CRE-1 is regulated by the WCC via VVD. Independent regulatory functions of VVD influencing cellulolytic efficiency include regulation of oxidative depolymerization, while for WC-1 and WC-2 these functions are most obvious in regulation of specific ribosomes. The WCC moreover specifically targets amino acid metabolism. Since the effect of the light response machinery is likely to be indirect, their output may involve additional target transcription factors.

Specific cellulase activity was slightly higher in all three photoreceptor mutants early in utilization of cellulose compared to WT (28 and 40 hrs), but the Δ*vvd *mutant showed significantly more cellulase activity after 5 days of growth on Avicel. These data suggest that, over time, WC-1 and WC-2 promote cellulose utilization, while VVD negatively antagonizes it. Consistent with this hypothesis, the Δ*wc-1 *and *Δwc-*2 mutants showed significant decrease in expression levels for the major predicted cellulase and hemicellulase genes. In case of Δ*vvd*, both the enrichment of carbohydrate metabolism genes, as well as the beneficial effect of several genes on cellulose degradation, as revealed by mutant analysis, indicate that the reason for enhanced cellulose degradation by the Δ*vvd *mutant lies at least in part in increased expression level of hydrolytic enzymes. From studies of circadian/light regulation by photoreceptors in *N. crassa*, it has been proposed that a hierarchical cascade of transcription factors regulated by WCC mediate light and circadian rhythm signaling networks, which in turn regulate 5-20% of the genes in the *Neurospora *genome [7,8,73]. The activation of light-induced gene expression by WCC is transient, so that levels of mRNA decrease in continuous light to reach a steady state level [74]. The attenuation of light-induced gene regulation by WCC is termed photoadaptation and requires VVD [20,37]. Photoadaptation occurs via light-dependent physical interaction of VVD and WC-1 LOV domains [23], resulting in inactivation of the WCC. Thus, in the Δ*vvd *mutant, photoadaptation is not functioning, which results in increased and prolonged expression levels of genes regulated by WCC, including those encoding cellulolytic enzymes and genes involved in energy metabolism (Figure 8).

Induction of cellulolytic genes and enzyme activity requires release from carbon catabolite repression (CCR), which is mediated in part by CRE-1 in filamentous fungi, which directly inhibits expression of genes encoding important plant cell wall degrading enzymes [43,70,75-77]. CRE-1 is also a target of the WCC [7]. In this respect, the negative effect of all three photoreceptors on transcription of *cre-1 *after 40 hours (Additional file 2: Dataset 1) indicates that their regulatory contribution to CCR is mediated by the WCC via VVD and not (only) subject to photoadaptation (Figure 8). Besides release from CCR, optimal expression of genes encoding plant cell wall degrading enzymes and cellulolytic activity requires the presence of inducer molecules. Our data suggests that the WCC regulates genes involved in plant cell wall degradation indirectly rather than directly, perhaps via activation of a target transcription factor (Figure 8). Consistent with this model is the observation that one of the transcription factors identified as a direct target of the WCC is required for growth of *N. crassa *on cellulose (Glass et al., unpublished results). In addition, regulatory output of VVD includes also WCC-independent targets, such as genes involved in oxidative depolymerization, which are likely to be important contributors to extracellular cellulase activity.

Our data do not support a model for the regulation of plant cell wall degradation by photoreceptors in which they exert their function - at least in part - via VVD, which is their regulatory target. The effects of lack of VVD considerably differ from those caused by deletion of *wc-1 *or *wc-2*. Moreover, VVD, WC-1 and WC-2 also individually have distinct targets, hence suggesting a complex regulation scheme, which can respond to different physiological cues. An interrelationship of the light response pathway with pathways sensing nutritional signals has been shown in *T. reesei *[9,78]. The distinct function of each of the three photoreceptors could reflect integration of multiple environmental signals with the light signal that enable optimal growth on cellulose.

Chen and coworkers [8] found genes involved in metabolic processes to be predominantly among the late light regulated genes (LLRGs), thus WCC may contribute to maintenance of appropriate energy supply in response to environmental conditions and substrate availability. Notably, energy metabolism, including glycogen metabolism, is positively regulated by all three photoreceptors and is likely to be perturbed in the mutants. The early enhanced extracellular cellulase activity and the considerably lower cellulase activity produced by Δ*wc-1 *and Δ*wc-2 *compared to the wild type after five days of growth (Figure 1) may partly be a reflection of a perturbation of energy metabolism in the Δ*wc-1 *and Δ*wc-*2 mutants. Consistent with this hypothesis, the lack of WC-1 or WC-2 also causes distinctly altered transcription patterns of numerous ribosomal genes and decreased transcription of genes involved in amino acid metabolism. An interconnection between cellulase activity and amino acid metabolism has been shown in *T. reesei *[48]; our data supports the conservation of this interconnection in *N. crassa*. The fact that the major regulator of starvation for amino acids, CPC-1, has 443 direct or indirect targets in *N. crassa *[32] reflects the importance of this regulator; a Δ*cpc-1 *mutant showed reduced ability to grow on cellulose and reduced cellulolytic activity (Figure 7). The integration of a response to amino acid starvation with enhanced production of enzymes degrading extracellular substrates could be a useful strategy to acquire the missing nutrients present in plant cells besides cellulose. Interestingly, genes encoding ribosomal proteins are coordinately regulated during carbon source shifts [79-81] and this response is not mediated by CRE-1 [82]. Since photoreceptors have been shown to control sensitivity to and utilization of carbon sources [83] the significant enrichment of ribosomal gene regulation likely reflects altered or decreased sensitivity to the extracellular carbon source. The slightly enhanced cellulase activity during early growth of Δ*wc-1 and *Δ*wc-2 *may therefore be a consequence of the attempt to compensate for this defect by adjustment of the ribosome pool [84,85].

Our study shows that the adjustment of the expression of cellulolytic genes triggered by light and the photoreceptors is sophisticated (Table 2). We evaluated the celluloytic phenotype of a number of genes with either predicted roles in plant cell wall degradation, or which were differentially regulated in the mutants. The majority of the mutants did not display cellulolytic activity that was significantly different from WT. However, it has been shown that deletion of a single cellulase gene does not necessarily causes decrease the capacity to degrade cellulose [31] and that additional genes contribute to the efficiency of the cellulase mixture [40]. In fact, Tian and coworkers [31] showed that deletion of certain cellulase genes can result in an increase in cellulase activity, suggesting compensatory mechanisms are occurring. We predict that the complex machinery of *N. crassa *allows for a sophisticated fine-tuning plant cell wall degrading enzyme repertoire in response to environmental conditions, which is also reflected in the regulatory patterns revealed in the photoreceptor mutants.

Although the transcriptional regulatory effects on predicted cellulolytic genes in the Δ*wc-1 *and Δ*wc-2 *mutants were similar, specific effects were also observed. For example, the Δ*wc-2 *mutant showed decreased transcription of six additional cellulolytic genes (Table 1). The importance of WC-2 for interaction of WC-1 with FRQ [86] may be one reason for the more severe effect on transcriptional regulation that we observed in the Δ*wc-2 *mutant. Similarly, data from *T. atroviride *showed that over-expression of *brl2 *(the homologue of *wc-2*) exerted a stronger and partially contrary effect to over-expression of *brl1 *(homologue of *wc-1*) [87]. The fact that the (direct and indirect) targets of WCC upon growth on cellulose as revealed in this study, only show a relatively small overlap with those identified by Smith and coworkers [7] confirms that WC-1 and WC-2 are important factors for the interconnection between light response and regulation of carbon source utilization. These transcription factors can consequently be expected to have carbon source dependent functions in addition to their function in light response and circadian rhythmicity as was also suggested for *brl1 *and *brl2 *in *T. atroviride *[83]. The identification of direct targets of WC-1, WC-2 and the WCC under lignocellulolytic conditions is an important avenue for future studies.

## Conclusions

In this study aimed at elucidation of the molecular mechanisms causing light modulated cellulase gene expression, we showed that this phenomenon is conserved between *N. crassa *and *T. reesei*, and most probably also in other ascomycete species. In summary, all three photoreceptors of *N. crassa *execute distinct roles in regulation of cellulase utilization and are hence crucial determinants of the interconnection between light response and carbon metabolism.

## Methods

### Strains and culture conditions

The wild type laboratory strain, FGSC 2489, used for the *N. crassa *genome project, as well as the deletion mutants FGSC11556 (Δ*vvd*), FGSC11712 (Δ*wc-1*) and FGSC11124 (Δ*wc-2*) obtained from the Fungal Genetics Stock Center (FGSC) [88] were used for microarray analysis [89]. Additional deletion mutants used for screening were obtained from the FGSC. Strains were inoculated onto slants containing Vogel's minimal medium [90] at 25°C for 7 days in constant light conditions (approx. 300 lux) to suppress synchronous gene expression associated with circadian rhythms. Conidia were harvested with water and inoculated into 50 ml of Bird's medium [91] with 2% (w/v) Avicel cellulose (Sigma) as sole carbon source in 250 ml Erlenmayer flasks at a final concentration of 10^6 ^conidia/ml. Cells were grown under constant light at 25°C for 28 or 40 hours at 200 rpm. Growth in race tubes was analyzed using Bird's medium without carbon source (control) and with 1% of sucrose or carboxymethylcellulose (CMC) as carbon source. Measurements were done in triplicates and hyphal extension was measured daily.

### Measurements of cellulase activity, biomass and secreted protein

For measurement of specific endo-1,4-β-glucanase (cellulase) activity in culture filtrates of wild type and mutant *N. crassa *strains, azo-CM-cellulose (article number S-ACMCL; Megazyme, Bray, Ireland) was used according to the manufacturer's instructions. Biomass accumulation after 28 or 40 hrs of growth in the presence of cellulose was determined as described earlier [25]. Briefly, mycelia including residual cellulose was harvested, squeezed dry with Whatman filter paper, frozen in liquid nitrogen, crushed and suspended in 5 ml of 0.1 N NaOH. After sonication for 2 minutes twice, samples were incubated for 3 hours at room temperature and centrifuged at 14000 g for 10 minutes. Protein content in the supernatant was determined using the Bio-Rad Protein Assay (Bio-Rad, Hercules, US), which reflects biomass content. At least two biological and two technical replicates were considered for calculation of results for every analysis.

### RNA isolation and qRT-PCR

Strains were grown under the conditions specified with the respective experiments and fungal mycelia were harvested by filtration and frozen in liquid nitrogen. Total RNA was isolated by using TRIzol reagent (Invitrogen Life Technologies), and purified by using the RNeasy kit (QIAGEN) according to the manufacturer's protocols. cDNA preparation for qRT-PCR was done using the RevertAid H^- ^cDNA Kit (No. K1631; Fermentas, Vilnius, Lithuania). qRT-PCR was performed using the iQ SYBR Green Supermix containing fluorescein (Bio-Rad, Hercules, USA) and the iCycler iQ5 Real-time PCR System (Bio-Rad). The *l6e *gene (NCU02702; primers: RT_NC_L6eF (5' CAGAAATGGTACCCTGCTGAGG 3') and RT_NC_L6eR (5' GCGGATGGTCTTGCGG 3')) was used as reference gene [29,92]. Primers used for analysis of *cbh-1 *(NCU07340) were RT_NC_cbh1F (5' CACCACCATCGAACAGCAC 3') and RT_NC_cbh1R (5' CAGTCTTGCCCTCACCGTAG 3') and for *cbh-2 *(NCU09680) RT_NC_cbh2F (5' CCCATCACCACTACTACC 3') and RT_NC_cbh2R (5' CCAGCCCTGAACACC 3'). Analysis of results was performed as described in [29] and for statistical analyses of expression ratios REST^© ^Software was used with a confidence interval of 95% [93].

### cDNA labeling for microarray analysis

For cDNA synthesis and labeling, the Pronto kit (catalog no. 40076, Corning) was used. Independent samples from two biological replicates were used. Briefly, cDNA was synthesized from a mixture containing 10 μg total RNA and oligo(dT)primer, ChipShot reverse transcriptase, and aminoallyl-deoxynucleoside triphosphate and incubated at 42°C for 2 h. The cDNA was purified by using ChipShot membrane columns. The dyes Cy3 and Cy5 (Amersham; catalog no. RPN5661) were incorporated into cDNA by adding Cy3 or Cy5 monofunctional N-hydroxy-succinimide ester dye to the cDNA solution for 1 h at 22°C. The cDNA was subsequently dried under vacuum and used for hybridization.

### Hybridization and image acquisition

For this analysis, *N. crassa *whole genome 70-mer oligonucleotide arrays were used as described earlier [33]. A GenePix 4000B scanner (Axon Instruments, CA) was used to acquire images and GenePix Pro6 software was used to quantify hybridization signals. Low-quality spots were flagged automatically by GenePix software and additionally each slide was expected manually.

### Experimental design and data analysis

We chose a closed-circuit design for microarray comparisons of WT and recombinant strains at the two different time-points (Additional file 1: Figure S3). Circuit designs for microarrays are statistically robust and improve resolution in identifying differentially regulated genes compared to designs for microarrays that use a universal reference [94]. Data analysis was performed essentially as described in [31-33]. Expression data has been submitted to GEO (http://www.ncbi.nlm.nih.gov/gds; Accession Number = GSE32871). For further analysis hybridized spots with a mean fluorescence intensity for at least one of the Cy3 and Cy5 dyes that was greater than the mean background intensity plus three standard deviations were scored, if less than 0.02% of the pixels were saturated.

The relative expression data of genes was estimated by the BAGEL software (34). BAGEL explores the ratio for all samples using a Markov Chain Monte Carlo (MCMC) approach in a Bayesian framework, and infers relative expression levels and statistical significance from the parameter values it samples. BAGEL also provides gene-by-gene 95% credible intervals, values in which 95% of the samples from the chain are bounded. Comparisons of gene expression levels in this study that were identified with at least 1.5 fold change at significant differences of the 95% credible level (p-value = 0.05), in WT versus mutant strains (Δ*wc-1*, Δ*wc-2 *and Δ*vvd*) at the conditions tested. Significant distance in gene expression was determined essentially as described in [31,32] using the PERL script "errorbar.pl" for expression differences as described in the text.

These inferred levels of gene expression were clustered using Hierarchical Clustering Explorer 3.5 (HCE; Human-Computer Interaction Lab, University of Maryland, College Park, USA) and applying default parameters (Euclidian Distance model).

The functional catalog FunCat created by MIPS [95] was mined to associate functional annotations with *Neurospora *genes http://mips.helmholtz-muenchen.de/genre/proj/ncrassa/Search/Catalogs/searchCatfirstFun.html. The statistically significant overrepresentation of gene groups in functional categories relative to the whole genome was determined with the MIPS online FunCat system using hypergeometric distribution for P value calculation http://mips.gsf.de/proj/funcatDB/help_p-value.html. We set the threshold for significant enrichment to P < 0.001.

## Competing interests

The authors declare that they have no competing interests.

## Authors' contributions

MS carried out experimental work and data analysis and drafted the manuscript. CG supervised microarray analysis and participated in data analysis. JS and DT participated in cultivation and analysis of cellulase expression. DT performed qRT-PCR analyses. MS and NLG collaborated in designing the study and writing the final version of the manuscript. All authors read and approved the final manuscript.

## Supplementary Material

Additional file 1 **Supplementary information**. Figure S1 - Profile of total secreted proteins in wild-type and photoreceptor mutants, Figure S2 - Analysis of hyphal extension rates, Figure S3 - Experimental design for microarray analysis of photoreceptor strains, Figure S4 - Transcript abundance of major cellulase genes upon cultivation on cellulose, Figure S5 - Transcript abundance of selected cellulse and hemicellulase genes upon growth on cellulose, Table S1 - Genes with a putative function related to fenton chemistry and in oxidative depolymerization of cellulose. Click here for file

Additional file 2 **Dataset 1**. Microarray data of *N. crassa *wild-type and photoreceptor mutants upon growth on cellulose. Click here for file

Additional file 3 **Dataset 2**. Functional category analysis of genes up- or down-regulated in photoreceptor mutants. Click here for file

Additional file 4 **Dataset 3**. Functional category analysis of genes assigned to coregulated clusters. Click here for file

Additional file 5 **Dataset 4**. Data on specific regulation by WC-1 and/or WC-2. Click here for file

Additional file 6 **Dataset 5**. Data on CPC-1 target genes regulated by WC-1 and WC-2. Click here for file
