# Supplementary material for: Unravelling the molecular basis for light modulated cellulase gene expression - the role of photoreceptors in Neurospora crassa
Source: BMC Genomics. 2012 Mar 31;13:127. doi: 10.1186/1471-2164-13-127 (PMC3364853; doi:10.1186/1471-2164-13-127)
Supplement: Additional file 1 — Supplementary information. Figure S1 - Profile of total secreted proteins in wild-type and photoreceptor mutants, Figure S2 - Analysis of hyphal extension rates, Figure S3 - Experimental design for microarray analysis of photoreceptor strains, Figure S4 - Transcript abundance of major cellulase genes upon cultivation on cellulose, Figure S5 - Transcript abundance of selected cellulse and hemicellulase genes upon growth on cellulose, Table S1 - Genes with a putative function related to fenton chemistry and in oxidative depolymerization of cellulose. [file 1471-2164-13-127-S1.PDF]

Supplementary figures and table

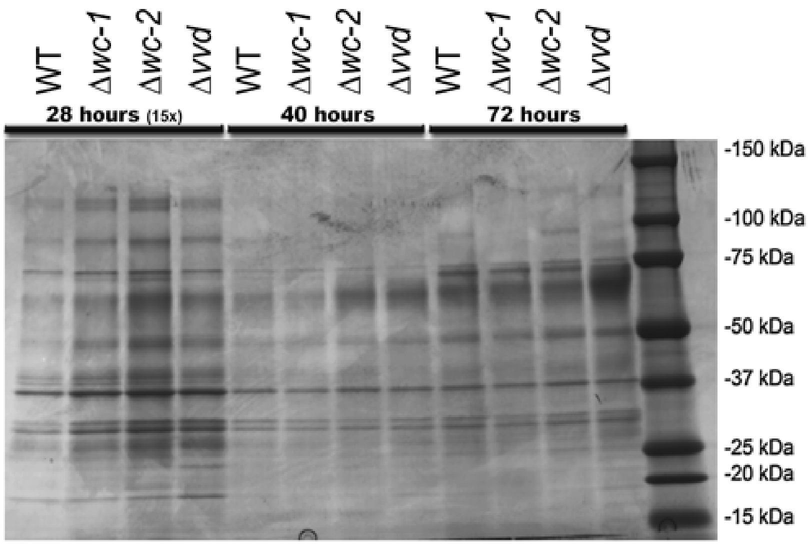

Supplementary Figure S1 **Profile of total secreted proteins in wild-type, Δ*vvd*, Δ*wc-1* and Δ*wc-2*.** Strains were grown on 2 % (w/v) Avicel cellulose and cultures were harvested at 28, 40 and 72 hours. Culture filtrates harvested after 28 hours were concentrated 15-fold.

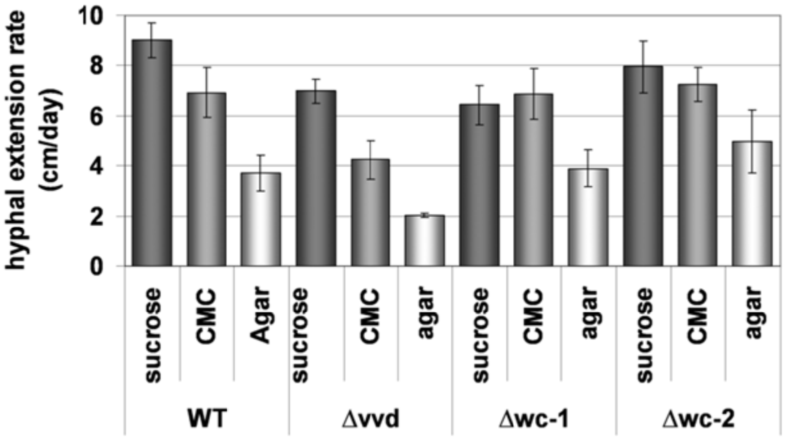

Supplementary Figure S2 **Analysis of hyphal extension rates** in race tubes upon growth on agar (control), or with sucrose (1 % w/v) or carboxymethylcellulose (1% w/v) as carbon source.

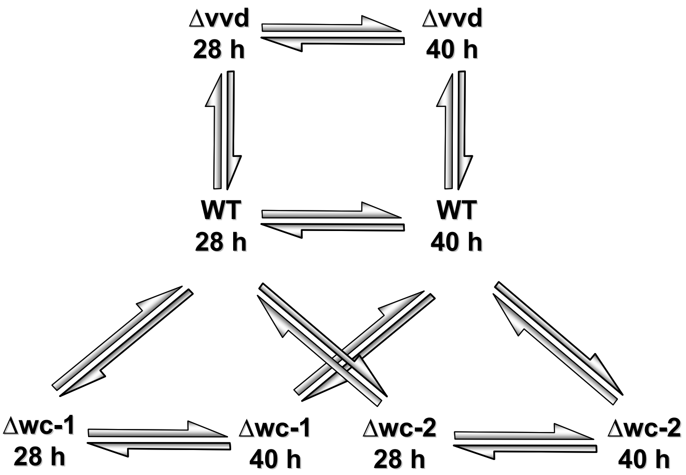

Supplementary Figure S3. **Experimental design for microarray analysis of photoreceptor strains**. A closed circuit design for microarray comparisons was used. Circuit designs are statistically robust and improve resolution in identifying differentially regulated genes compared to designs for microarrays that use a universal reference. Each arrow represents hybridization and the arrowhead points to Cy5-labeled cDNA.

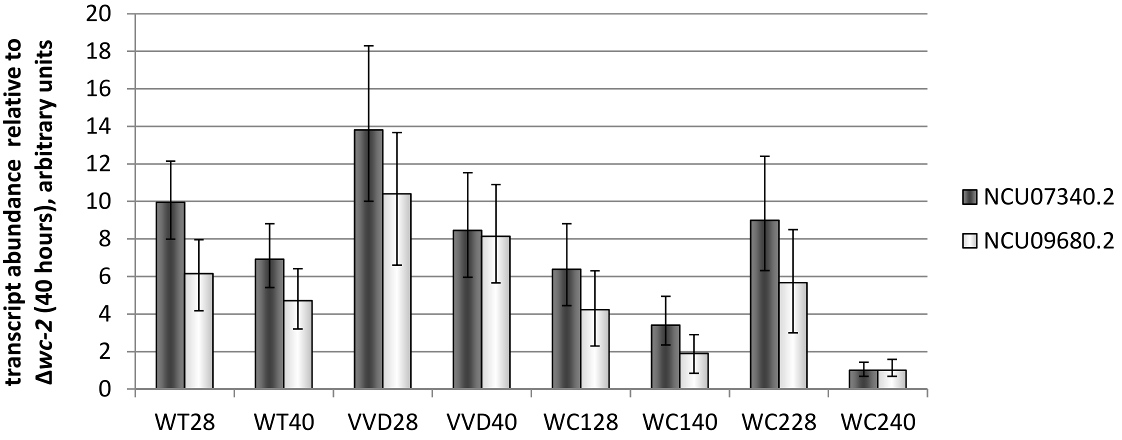

Supplementary Figure S4. **Transcript abundance of NCU07340 (encoding CBH-1) and NCU09680 (encoding CBH-2/GH6-2) upon cultivation on cellulose.** Microarray data showing WT after 28 (WT 28) or 40 (WT 40) hours of growth on cellulose as well as Δ*vvd* (VVD28 or VVD40), Δ*wc-1* (WC1 28 or WC1 40) and Δ*wc-2* (WC2 28 or WC2 40) under these conditions. Errorbars reflect error margins as calculated by BAGEL.

A

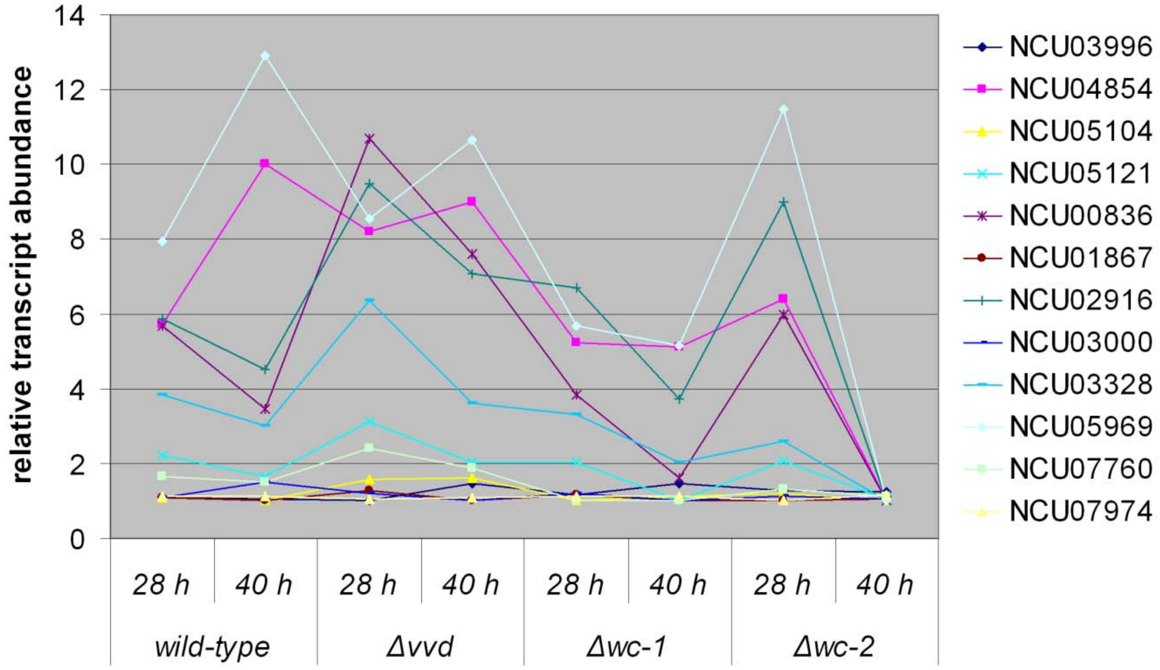

B

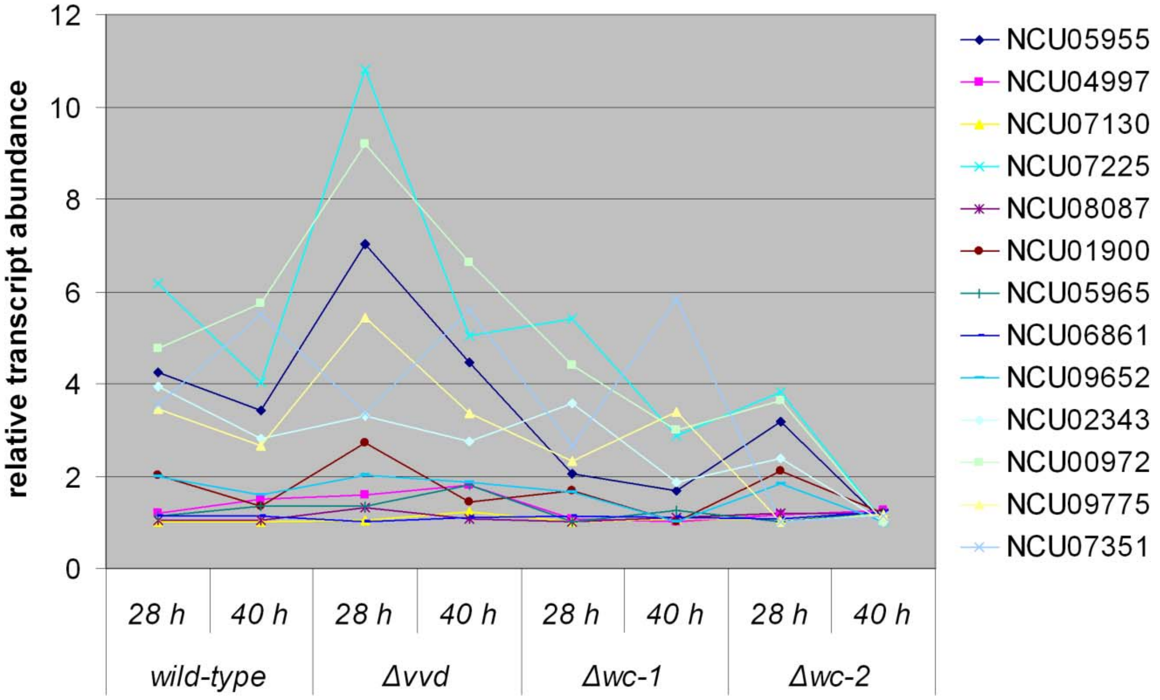

Supplementary Figure S5. **Transcript abundance in wild-type and mutant strains upon growth on cellulose for 28 or 40 hours.** (A) Cellulase genes not evaluated for their contribution to cellulase activity. (B) Hemicellulase genes not evaluated for their contribution to cellulase activity.

Supplementary Table S1. **Genes with a putative function related to fenton chemistry and in oxidative depolymerization of cellulose**

predicted function	locus ID
catalase	NCU08971
catalase	NCU05169
catalase	NCU00355
copper oxidase	NCU05770
copper SOD	NCU02133
copper SOD	NCU03013
ferric reductase	NCU02278
ferric reductase	NCU10775
ferric reductase	NCU00023
ferric reductase	NCU02009
ferric reductase	NCU02020
ferric reductase	NCU02110
ferric reductase	NCU08194
ferric reductase	NCU00829
ferric reductase	NCU00876
GMC oxidoreductase	NCU01853
GMC oxidoreductase	NCU07113
GMC oxidoreductase	NCU09798
GMC oxidoreductase	NCU04938
GMC oxidoreductase	NCU01193
GMC oxidoreductase	NCU09024
GMC oxidoreductase	NCU08977
iron permease	NCU03497
laccase	NCU05113
laccase	NCU09279
MFS quinate transporter	NCU05585
MFS quinate transporter	NCU00988
multicopper oxidase	NCU02201
multicopper oxidase	NCU05604
multicopper oxidase	NCU09279
multicopper oxidase	NCU04593
multicopper oxidase	NCU07920
multicopper oxidase	NCU05113
multicopper oxidase	NCU05042
multicopper oxidase	NCU00526
multicopper oxidase	NCU03498
multicopper oxidase	NCU04528
multicopper oxidase	NCU09023
quinate permease	NCU06026
quinone oxidoreductase	NCU04443
quinone oxidoreductase	NCU01419
quinone oxidoreductase	NCU04044
quinone oxidoreductase	NCU02948
